# Role of Baseline Antral Follicle Count and Anti-Mullerian Hormone in Prediction of Cumulative Live Birth in the First In Vitro Fertilisation Cycle: A Retrospective Cohort Analysis

**DOI:** 10.1371/journal.pone.0061095

**Published:** 2013-04-23

**Authors:** Hang Wun Raymond Li, Vivian Chi Yan Lee, Estella Yee Lan Lau, William Shu Biu Yeung, Pak Chung Ho, Ernest Hung Yu Ng

**Affiliations:** Department of Obstetrics and Gynaecology, Centre of Assisted Reproduction and Embryology, The University of Hong Kong, Queen Mary Hospital, Hong Kong; VU University Medical Center, The Netherlands

## Abstract

**Objective:**

This retrospective study determined for the first time the role of baseline antral follicle count (AFC) and serum anti-Mullerian hormone (AMH) level in the first in-vitro fertilisation (IVF) cycle in predicting cumulative live birth from one stimulation cycle.

**Methods:**

We studied 1,156 women (median age 35 years) undergoing the first IVF cycle. Baseline AFC and AMH level on the day before ovarian stimulation were analysed. The main outcome measure was cumulative live birth in the fresh plus all the frozen embryo transfers after the same stimulation cycle.

**Results:**

Serum AMH was significantly correlated with AFC. Both AMH and AFC showed significant correlation with age and ovarian response in the stimulated cycle and total number of transferrable embryos. Baseline AFC and serum AMH were significantly higher in subjects attaining a live birth than those who did not in the fresh stimulated cycle, as well as those attaining cumulative live birth. There was a significant trend of higher cumulative live birth rate in women with higher AMH or AFC. However, logistic regression revealed that both AMH and AFC were not significant predictors of cumulative live birth after adjusting for age and number of embryos available for transfer. Considering only one single predictor, the areas under the ROC curves for AMH (0.646, 95% CI 0.616–0.675) and age (0.648, 95% CI 0.618–0.677) were slightly higher than that for AFC (0.617, 95% CI 0.587–0.647) in predicting cumulative live birth. However, a model combining AMH (with or without AFC) and age of the women only classified an addition of less than 2% of subjects correctly compared to the model with age alone.

**Conclusion:**

Baseline AFC and serum AMH have only modest predictive performance on the occurrence of cumulative live birth, and may not give additional value on top of the women's age.

## Introduction

In assisted reproduction programmes, a number of parameters known as ovarian reserve markers, such as serum follicle stimulating hormone (FSH) concentration, antral follicle count (AFC) and serum anti-Mullerian hormone (AMH) concentration, are widely used to predict ovarian responses to gonadotrophin stimulation during in-vitro fertilization (IVF) treatment. These markers may help to decide on the initial dose and regimen of stimulation [1], [2]. Among the commonly used ovarian reserve markers, AFC and AMH provide the best performance in predicting both poor and excessive ovarian response [3], [4], [5]. However, most reports consistently showed a poor predictive value of these markers on pregnancy rate in the fresh IVF cycle [4], [6].

AMH, also known as Mullerian-inhibiting substance, is a dimeric glycoprotein that belongs to the transforming growth factor-beta family. It is involved in the regression of the Mullerian ducts during development of the male fetus [7]. In the adult female, AMH is exclusively produced by granulosa cells of preantral and small antral follicles, and has been shown to correlate excellently with the primordial follicle pool [8]. Hence it would serve as an ovarian reserve marker. Since AMH performs equally well, if not better, than AFC in predicting ovarian response and that it is both operator- and menstrual cycle-independent, there has been a growing trend to adopt AMH assay as the first-line ovarian reserve test [2], [9].

In modern-day assisted reproduction programmes, embryo cryopreservation has become an integral component. It allows the storage and subsequent usage of surplus good quality embryos in frozen-thawed embryo transfer (FET) cycles. Hence, to evaluate the outcome of an IVF cycle, it would be more logical to consider the cumulative live birth rate from the fresh and all FET cycles combined, instead of merely looking at the single fresh cycle outcome.

There have been limited data on the role of these ovarian reserve markers in predicting cumulative pregnancy or live birth rates in IVF programmes. A study looked at the use of AMH on Day 6 of ovarian stimulation in predicting cumulative ongoing pregnancy outcome [10]. Yet AMH levels can be altered after commencement of ovarian stimulation [11], [12], [13], and it may be of more practical value to study AMH level before commencement of stimulation, and to look at live birth as the outcome which would be what patients are ultimately concerned about.

Therefore, we conducted this retrospective analysis to evaluate the role of baseline AFC and AMH in the first stimulated IVF cycle in predicting the cumulative live birth from the stimulated cycle and all subsequent FET cycles derived from that stimulated cycle.

## Materials and Methods

### Subject selection

We reviewed all first IVF cycles carried out between January 2007 and December 2009 at the Centre of Assisted Reproduction and Embryology, The University of Hong Kong – Queen Mary Hospital, Hong Kong. Cycles carried out for pre-implantation genetic diagnosis or those using donor oocytes were excluded from this analysis. Clinical details of all treatment cycles were prospectively entered into a computerized database, which were retrieved for analysis.

### Ethics statement

An ethics approval was obtained from the Institutional Review Board of the University of Hong Kong/Hospital Authority Hong Kong West Cluster for this retrospective study to be carried out using existing patient data in an anonymous manner without requiring written consent from individual patients. The project did not involve any additional intervention or modification from the standard treatment.

### Stimulation cycle, AFC and AMH determination

All patients attended the clinic for blood test and pelvic ultrasound examination at the beginning of the IVF treatment cycle before commencing ovarian stimulation. Transvaginal scanning was performed using a 5 MHz vaginal probe (Voluson 730®, GE Healthcare, Wisconsin, USA) to determine AFC (2–9 mm) in both ovaries. Blood was taken for routine oestradiol (E2) assay, and residual serum samples were stored at −20°C. For the current study, these archived serum samples were retrieved and assayed for AMH using the AMH Gen II ELISA kit (Beckman Coulter, Webster, TX, USA; catalogue number A79765). The assay has a sensitivity of 0.08 ng/ml (0.6 pmol/l), and intra- and inter-assay coefficients of variation of less than 5.4 and 5.6%.

All subjects were treated either with the long GnRH agonist protocol or the GnRH antagonist protocol for pituitary down-regulation, with the latter mostly for selected patients with poor ovarian reserve. In the long GnRH agonist protocol, the women received buserelin (Suprecur®, Hoechst, Frankfurt, Germany) nasal spray 150 µg four times a day starting from the mid-luteal phase of the cycle preceding the treatment cycle, and received human menopausal gonadotrophin (HMG) or recombinant FSH for ovarian stimulation after return of a period. In the GnRH antagonist protocol, after confirming a basal serum oestradiol level, ovarian stimulation was started. The women received ganirelix or cetrorelix 250 µg daily starting from the sixth day of stimulation. The initial dose of stimulation was determined according to the baseline AFC (AFC≥15: 150 IU per day; AFC between 6 and 14: 300 IU for the first two days followed by 150 IU daily; AFC ≤5: 300–450 IU for the first two days followed by 225 IU daily). Human chorionic gonadotrophin (hCG) (Pregnyl® 5000 or 10000 units or Ovidrel® 250 µg) was given when the mean diameter of the leading follicle reached 18 mm and there were at least 3 follicles reaching a mean diameter of 16 mm or above, followed by transvaginal ultrasound-guided oocyte retrieval 36 hours later. Fertilisation was carried out in-vitro either by conventional insemination or intracytoplasmic sperm injection (ICSI) depending on semen parameters.

Subjects were allowed to have replacement of at most two embryos two days post-fertilisation. Embryo transfer (ET) was performed under transabdominal ultrasound guidance using a soft catheter (Sydney IVF Embryo Transfer Catheter®, Cook, Indiana, USA). Fresh embryo transfer would be cancelled and all the embryos with good quality were cryopreserved on day 2 after the retrieval if the subject had symptoms suggestive of ovarian hyperstimulation syndrome (OHSS) or serum E2 concentration on the day of hCG injection was >20,000 pmol/L.

### Cryopreservation and frozen-thawed embryo transfer (FET)

Embryos were graded based on the number/regularity of blastomeres and the degree of fragmentation [14]. Surplus embryos of grades 1 to 4 were frozen on the day of ET, whereas embryos with poor quality (Grades 5 and 6) were discarded. Cryopreservation was performed by a slow freezing protocol using a programmable freezer (Planer Products Ltd.; Sunbury-On-Thames, UK) in straws of two. The details of the freezing and thawing protocols were reported previously [15]. The frozen embryos were thawed on the morning of FET. Embryos were discarded if more than 50% of the original blastomeres were lysed or degenerated upon thawing. Frozen-thawed embryos were transferred in natural cycles in ovulatory subjects. LH surge was determined by serial blood tests, and FET was performed on the third day after LH surge. For anovulatory subjects, FET was carried out in either clomiphene-induced or hormone replacement cycles. A maximum of two frozen embryos were allowed to be transferred in any one FET cycle.

### Pregnancy outcome

A urine pregnancy test was done 16 days after embryo transfer. Pregnant women were offered an ultrasound examination 10–14 days later to confirm intrauterine pregnancy and the number of gestational sacs present. Pregnancy outcome was traced from all pregnant women by postal questionnaire or by phone.

### Statistical analysis

The primary outcome measure was the cumulative live birth in the fresh and all FET cycles combined following the same index stimulation cycle. The age of the women used in analysis referred to the time of starting ovarian stimulation. Non-normally distributed continuous variables were expressed as median (interquartile range) unless otherwise stated. Continuous and categorical variables were compared between groups using Mann-Whitney test and Fisher's Exact test respectively. Logistic regression analysis was used to examine factors predicting cumulative live birth, and receiver-operator characteristic (ROC) curves were compared for different ovarian reserve markers in predicting cumulative live birth. Statistical analysis was carried out using the Statistical Program for Social Sciences (SPSS Inc., Version 15.0, Chicago, U.S.A.) and MedCalc (Version 12, Belgium). The two-tailed value of P <0.05 was considered statistically significant.

## Results

Of the 1,156 subjects who underwent the first IVF cycle during the study period, 1,050 (90.8%) were treated on the long GnRH agonist protocol, and the remaining 106 (9.2%) on the GnRH antagonist protocol. The age and body mass index (BMI) [median (interquartile range)] of our subjects were 35 (33–38) years and 21.2 (19.6–23.1) kg/m^2^ respectively. The cause of subfertility included male factor (687; 59.4%), tubo-peritoneal factor (159; 13.8%), endometriosis (54; 4.7%), anovulation (8; 0.7%), unexplained (62; 5.4%) and mixed causes (186; 16.0%).

### Correlation of AFC and AMH with other demographic and clinical characteristics

There was a significant correlation between serum AMH and AFC (R = 0.780, p<0.001). As shown in Table I, both serum AMH and AFC were significantly correlated with the women's age, duration of stimulation, total dose of gonadotrophin used, number of mature-sized follicles (≥16 mm), peak serum E2 concentration, number of oocytes retrieved and transferrable embryos obtained (for fresh transfer and/or freezing) and number of top quality embryos (p<0.001 for all). There was no significant correlation between serum AMH or AFC with BMI.

**Table 1:** Correlation of AMH and AFC with ovarian stimulation parameters in fresh stimulation cycle.

Parameter	Serum AMH		AFC	
	Correlation coefficient	p-value	Correlation coefficient	p-value
Age of women	−0.321	<0.001*	−0.335	<0.001*
Body mass index	0.001	0. 969	0.035	0.238
Duration of stimulation	−0.232	<0.001*	−0.224	<0.001*
Total dose of gonadotrophin	−0.590	<0.001*	−0.683	<0.001*
No of follicles > = 16 mm	0.548	<0.001*	0.449	<0.001*
Peak serum oestradiol	0.529	<0.001*	0.410	<0.001*
No. of oocytes retrieved	0.629	<0.001*	0.527	<0.001*
No. of transferrable embryos	0.438	<0.001*	0.364	<0.001*
No. of top quality embryos#	0.341	<0.001*	0.321	<0.001*

* statistically significant (p<0.005).

#defined as number of blastomeres ≥4 with grading 1 or 2 (data available in 593 subjects only).

### AFC, AMH and live birth in the fresh cycle

There were 1,026 subjects (88.8%) who had fresh ET. Among them, 510 (49.7%) had a positive pregnancy test in the fresh cycle, and 383 (37.3%) attained live birth. Among those who were pregnant, there was no significant difference (p>0.05) in AFC and serum AMH between those who miscarried and those who had live birth. The clinical and demographic characteristics of subjects with or without a live birth in the fresh cycle are compared in Table II. Subjects who attained a live birth were significantly younger (median 34.0 vs 36.0 years), had significantly higher AFC (median 10.0 vs 9.0) and serum AMH [median 3.27 ng/ml (23.3 pmol/l) vs 2.64 ng/ml (18.8 pmol/l)], and more of them had double embryo transfer (94.3% vs 82.0%). There were no statistically significant differences in BMI between subjects who had live birth in the fresh cycle or not, nor in the distribution of the cause of subfertility.

**Table 2:** Comparison of demographic and clinical characteristics of subjects with or without achieving live birth in the fresh IVF cycle.

Parameter	Subjects achieving a live birth in fresh IVF cycle (n = 383)	Subjects not achieving a live birth in fresh IVF cycle (n = 643)	P value
Age (years)	34.0 (32.0–37.0)	36.0 (34.0–38.0)	<0.001*
Body mass index (kg/m^2^)	21.0 (19.5–23.2)	21.2 (19.6–23.1)	0.927
Serum AMH (ng/ml)	3.27 (1.75–5.65)	2.64 (1.32–4.69)	<0.001*
Antral follicle count	10.0 (6–15)	9.0 (5–13)	0.001*
Cause of subfertilityMale factorTuboperitonal factorEndometriosisAnovulationUnexplainedMixed factors	236 (61.6%)49 (12.8%)19 (5.0%)6 (1.6%)19 (5.0%)54 (14.0%)	371 (57.7%)98 (15.2%)25 (3.9%)1 (0.2%)41 (6.3%)107 (16.7%)	0.056(Fisher's Exact test)
No. of embryos replacedOneTwo	22 (5.7%)361 (94.3%)	116 (18.0%)527 (82.0%)	<0.001 (Fisher's Exact test)

Continuous data are expressed as median (interquartile range).

* statistically significant.

Mann-Whitney U test unless otherwise stated.

Binary logistic regression using the enter method was used to analyse the prediction on live birth in the fresh IVF cycle by the women's age, AFC, serum AMH and number of embryos replaced. The women's age and the number of embryos replaced, but not AFC or serum AMH, were the significant factors which independently predicted the likelihood of a live birth in the fresh cycle (Table IIIa).

**Table 3:** Binary logistic regression analysis of factors for prediction of (a) a live birth in the fresh IVF cycle and (b) a cumulative live birth from the fresh plus all frozen-thawed transfer cycles combined after the same index stimulation cycle.

(a) Factors to predict a live birth in the fresh IVF cycle	B	Exp(B), 95% CI	P-value
AFC	−0.002	0.998, 0.972–1.025	0.880
AMH	0.020	1.020, 0.966–1.077	0.470
Age	−0.122	0.885, 0.849–0.923	<0.001*
Number of embryos transferred	1.236	3.441, 2.124–5.577	<0.001*

(b) Factors to predict a cumulative live birth	B	Exp(B), 95% CI	P-value
AFC	−0.004	0.996, 0.968–1.025	0.791
AMH	0.003	1.003, 0.995–1.012	0.441
Age	−0.129	0.879, 0.841–0.918	<0.001*
Total number of transferrable embryos	0.259	1.295, 1.228–1.366	<0.001*

* statistically significant.

**Figure 1:**
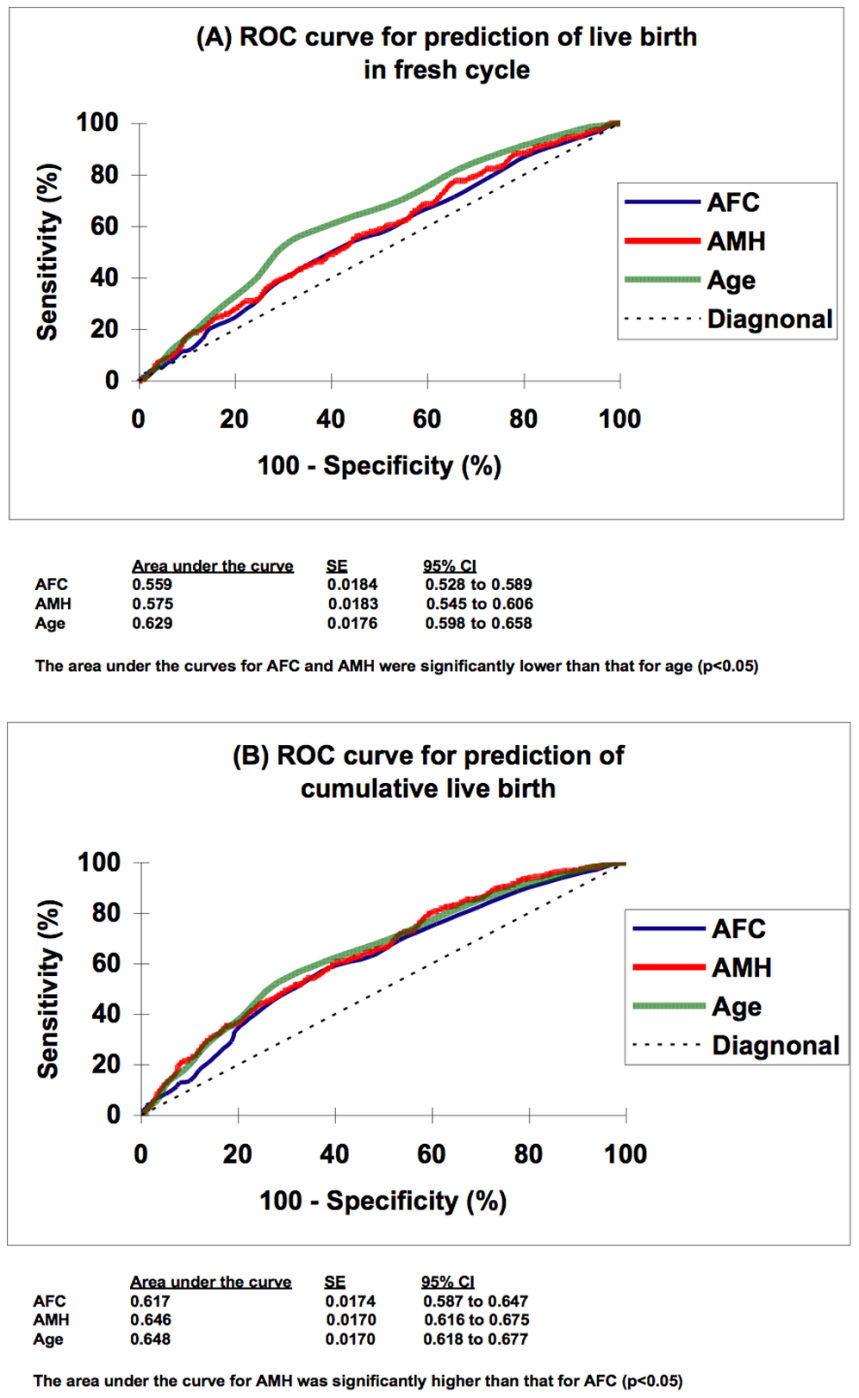
Receiver operator characteristic (ROC) curve analysis. Panal (A) shows the ROC curves of antral follicle count (AFC), serum AMH concentration and age of the women in predicting live birth from the fresh stimulation cycle, while Panal (B) shows those in predicting cumulative live birth from fresh and all frozen embryo transfers after the same index stimulation cycle.

Figure 1A shows the ROC curve analysis of AFC and serum AMH in predicting a live birth in the fresh cycle. The area under the curve (AUC) for AFC and serum AMH were 0.559 (95% CI 0.528–0.589) and 0.575 (95% CI 0.545–0.606) respectively, which were both inferior (p<0.05, Delong analysis) to that for age of the women (0.629, 95% CI 0.598–0.658).

### AFC, AMH and cumulative live birth

Among the subjects included in this study, 1,038 (89.8%) had completed replacement of all available embryos and with a live birth outcome available. Of them, 558 (53.8%) attained live birth cumulatively from the fresh plus all FET cycles after the same index IVF cycle.

Table IV depicts the demographic and clinical characteristics of subjects who had a cumulative live birth and those who did not. Subjects who attained a cumulative live birth were significantly younger (median 34.0 vs 36.0 years), had significantly higher AFC (median 11.0 vs 8.0) and serum AMH [median 3.7 ng/ml (26.4 pmol/l) vs 2.3 ng/ml (16.4 pmol/l), and had significantly higher number of transferrable embryos (median 5 vs 2). The minimum values of AFC and serum AMH in subjects who attained a cumulative live birth were 0 and 0.15 ng/ml (1.1 pmol/l) respectively (vs 0 and <0.08 ng/ml (<0.6 pmol/l) respectively in those without a cumulative live birth).

**Table 4:** Comparison of demographic and clinical characteristics of subjects with or without achieving a cumulative live birth from the fresh plus all the frozen-thawed embryo transfer cycles combined after the same index stimulation cycle.

Parameter	Subjects achieving a cumulative live birth (n = 558)	Subjects not achieving a cumulative live birth (n = 480)	P value
Age (years)	34 (32–37)	36 (34–38)	<0.001*
Body mass index (kg/m^2^	21.1 (19.5–23.1)	21.2 (19.7–23.2)	0.426
Serum AMH (ng/ml)	3.7 (1.9–6.1)	2.3 (1.1 6–4.2)	<0.001*
Antral follicle count	11 (7–16)	8 (5–12)	<0.001*
Cause of subfertilityMale factorTuboperitonal factorEndometriosisAnovulationUnexplainedMixed factors	348 (62.4%)69 (12.4%)23 (4.1%)7 (1.3%)31 (5.6%)80 (14.2%)	274 (57.1%)77 (16.0%)22 (4.6%)0 (0%)27 (5.6%)80 (16.7%)	0.048* (Fisher's Exact test)
Total number of transferrable embryos	5 (3–8)	2 (2–4)	<0.001*

Continuous data are expressed as median (interquartile range).

* statistically significant.

Mann-Whitney U test unless otherwise stated.

**Figure 2:**
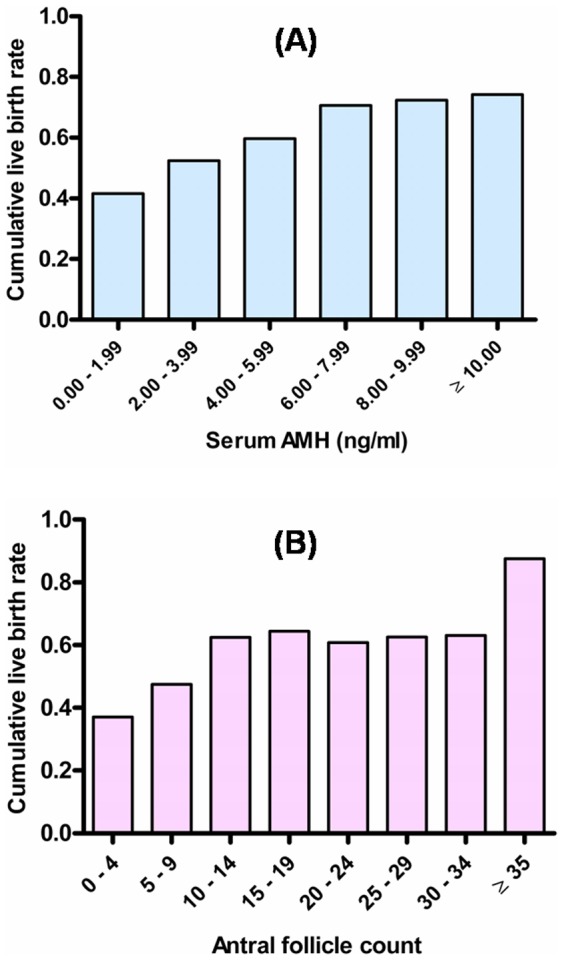
Cumulative live birth rate stratified according to serum AMH and antral follicle count (AFC). Panal (A) shows the association between serum AMH level and cumulative live birth rate from the fresh plus all frozen embryo transfer cycles combined, whereas Panal (B) shows that between AFC and cumulative live birth rate. There was a significant trend of increase across the bars from the lower to the higher ends of AMH and antral follicle count (p<0.001 for both, χ^2^ test for trend).

Figure 2 shows the cumulative live birth rate in subjects categorised according to baseline AMH and AFC. There was an overall significant trend of a higher cumulative live birth rate in subjects with higher serum AMH levels or AFC compared to those at the lower end (p<0.001 for both AMH and AFC, χ^2^ test for trend).

When the women's age, AFC, serum AMH and total number of transferrable embryos derived from the index IVF cycle were entered into the binary logistic regression model using the enter method, the women's age and total number of transferrable embryos, but not AFC or AMH, were significant independent factors in predicting a cumulative live birth (Table IIIb).

We further analysed the predictive performance of AFC and AMH in additional to age of the women versus age alone on cumulative live birth. (Table V). The addition of AMH, but not AFC, to age provided additional predictive value compared to using age alone. However, although statistically significant (p<0.05, Delong analysis), the model combining the women's age and AMH, with or without AFC, only respectively classified an additional 1.7% or 1.9% of subjects correctly compared to the model with age alone.

**Table 5:** Comparison of AMH, AFC in addition to age and age only in predicting cumulative live birth by logistic regression analysis.

Model	Predictors	OR (95% CI)	Classification rate	#AUC (95% CI)
A	Age	0.86 (0.81–0.92)	63.6%	a0.69 (0.64–0.74)
	AMH	1.02 (0.98–1.07)		
	AFC	1.06 (0.97–1.15)		
B	Age	0.86 (0.80–0.92)	63.8%	b0.69 (0.63–0.74)
	AMH	1.09 (1.02–1.16)		
C	Age	0.86 (0.81–0.92)	61.7%	c0.68 (0.63–0.73)
	AFC	1.04 (1.01–1.07)		
D	Age	0.84 (0.79–0.90)	61.9%	0.66 (0.60–0.71)

#The AUCs were derived from the logistic regression models.

a *p* = 0.026 compared with AUC for model A and AUC for model D.

b *p* = 0.018 compared with AUC for model B and AUC for model D.

c *p* = 0.083 compared with AUC for model C and AUC for model D.

Figure 1B shows ROC curve analysis of AFC, serum AMH and age in predicting a cumulative live birth. The AUC for serum AMH and age were 0.646 (95% CI 0.616–0.675) and 0.648 (95% CI 0.618–0.677) respectively, which were both significantly higher (p<0.05) than that for AFC (0.617, 95% CI 0.587–0.647).

## Discussion

In assisted reproduction programmes, ovarian reserve markers such as FSH, AFC, and more recently AMH, have been widely used for predicting ovarian response and prognostic counseling. Recent systematic reviews have suggested that AFC and AMH were the best predictors of excessive and suboptimal ovarian response and both had comparable performance in this regard [4], [5]. AMH has the additional benefits of being not operator-dependent and having negligible inter-cycle variations [16], and hence has gained increasing favour in its clinical applications [2], [9]. However, the majority of reported studies consistently demonstrated that both AFC and AMH had poor performance in predicting pregnancy outcome in IVF cycles [4], [6].

Nonetheless, most of the reported studies evaluated the pregnancy or live birth outcome in the fresh IVF cycle only. In modern assisted reproduction programmes, cryopreservation of surplus embryos with subsequent transfers in thawed cycles has become an integral part. It has been estimated that in programmes which incorporate embryo cryopreservation, up to 42% of all conceptions could be derived from FET [17]. As there is a recent trend in reducing the number of embryos replaced to reduce the risk of multiple pregnancy, the number of live births from the stimulated cycle only is not a good indicator of outcome of treatment. Hence, we feel that it would be more meaningful to study the cumulative live birth per stimulated cycle as the treatment outcome. This would take into account the outcomes in the transfer of fresh as well as frozen embryos derived from the same index stimulation cycle, the latter of which should not be ignored. There was one study which reported that serum AMH on day 6 of stimulation was significantly higher in subjects attaining cumulative ongoing pregnancy than those who did not [10]. However, it has been reported that serum AMH is suppressed during ovarian stimulation compared to baseline level under both GnRH agonist and antagonist protocols [11], [12], [13]. Practically, measurement of the baseline AMH level before stimulation would be of more relevance for either prognostic prediction or individualization of gonadotrophin dosing. To our knowledge, the current study is the first to report on the role of baseline serum AMH and AFC in the index stimulation cycle in predicting cumulative live birth outcome so derived in a large cohort.

Our results demonstrated that women who attained a cumulative live birth had significantly higher serum AMH and AFC at baseline before ovarian stimulation. However, the predictive performance of both parameters on the absolute occurrence of cumulative live birth was only modest and not better than age as demonstrated in the ROC curves. This is obviously comprehensible by observing the trend of cumulative live birth rate over serial increase in serum AMH or AFC as shown in Figure 2. There was a gradual increase in cumulative live birth rate in women with higher AMH or AFC, and yet it varied over a continuum with no clear cut-off where the cumulative live birth rate could show an abrupt change. Hence, it would be impossible for any set value of AMH or AFC to predict the all-or-none occurrence of a cumulative live birth.

Hypothetically, women with higher AMH or AFC have better ovarian reserve and hence higher oocyte yield and a larger potential pool of good quality embryos to be selected for transfer. This was supported by our findings that subjects who attained cumulative live birth had higher number of transferable embryos, and that the latter was an independent factor predicting cumulative live birth in logistic regression analysis. This is in line with a recent study which suggested that the total number of good-quality embryos and total number of transferable embryos were the two most important predictors of cumulative clinical pregnancy, particularly in women aged below 40 years [18]. Analysis of our data showed that the cumulative live birth rate increased with increasing number transferrable embryos up till 15, after which it plateaued (data not shown). The same has been recently reported on a large cohort of 400,135 IVF treatment cycles, in which the live birth rate in the fresh IVF cycle correlated positively with the number of retrieved oocytes up till 15, after which it plateaued and actually declined after going beyond 20 [19], though the study did not take into account the FETs. Therefore, at the higher end of embryo yield, further increase in embryo number does not necessarily mean better live birth outcome because the occurrence of live birth is subjected to influence by other factors as well, such as embryo quality and endometrial receptivity.

With regard to endometrial receptivity, women with high AFC and/or serum AMH levels are at increased risk of ovarian hyperstimulation, and the resultant higher peak E2 levels could have detrimental effects on endometrial receptivity [20], [21]. This may offset the potentially favourable implication of higher ovarian response in the fresh stimulated cycle. Embryo quality is another major issue, which seemingly cannot be predicted by either AMH or AFC, as illustrated by previously reported studies which revealed no consistent correlation between AMH and embryo morphology nor aneuploidy rate [22], [23], [24]. Hence all these parameters do not seem to have good prediction on the qualitative aspect of embryo competence. Probably there is no single factor which can precisely predict cumulative live birth outcome, which is actually determined by the complicated interplay of multiple factors. Indeed, among our subjects who had serum AMH <0.5 ng/ml (<3.6 pmol/l), a cumulative live birth rate of 27% could still be attained, and in those with AFC ≤1, again a cumulative live birth rate of 22% was observed (data not shown). Moreover, a cumulative live birth did occur with a minimum baseline AMH level of 0.15 ng/ml (1.1 pmol/l) and AFC of zero.

We also analysed the value of adding AFC and AMH on top of the women's age in the prediction model for cumulative live birth. Although statistically significant, the combined model with the women's age and AMH (with or without AFC) classified an addition of less than 2% of subjects correctly compared to the model with age alone. This very modest improvement in the prediction may not be very meaningful clinically.

Based on our results, we would recommend that it is not worthwhile to use any of the ovarian reserve markers including serum AMH or AFC as the basis to exclude subjects from attempting assisted reproduction treatments, and when used for prognostic counselling the women should understand the limitations and imprecision of these indices in anticipating the treatment outcome.

Inherent to the retrospective design, our results might have been subjected to verification bias, as the gonadotrophin dosing regimen has been preselected based on AFC in our protocol. We have repeated the analysis on the subgroup of subjects stimulated with an average daily dose of 225 IU or more, which presumably gave maximal stimuation. There was no significant difference in the areas under the ROC curve for AMH, AFC and age in predicting cumulative live birth (data not shown).

The strength of our study was the inclusion of a large cohort of subjects over a wide age range from 22 to 45 years with diversified ovarian reserve to enhance generalisability. Although some of the subjects had not completed replacement of all available frozen embryos and were hence excluded from the cumulative live birth analysis, they did not differ significantly from the included subjects in sociodemographic parameters.

In conclusion, our results confirmed a good correlation of baseline serum AMH and AFC with ovarian response parameters in IVF treatment. Baseline AFC and serum AMH were significantly higher in subjects attaining cumulative live birth, and there was a significant trend of increase in cumulative live birth rate in women with higher AMH or AFC. However, the predictive role of AFC and AMH on absolute occurrence of cumulative live birth was only modest and may not give much additional value on top of the women's age.
